# The importance of central corneal thickness measurements and decision making in general ophthalmology clinics: a masked observational study

**DOI:** 10.1186/1471-2415-8-1

**Published:** 2008-01-20

**Authors:** Ashish A Patwardhan, Mohammad Khan, Susan P Mollan, Paul Haigh

**Affiliations:** 1 Department of Ophthalmology, Shrewsbury and Telford Hospitals NHS Trust, Shrewsbury, UK

## Abstract

**Background:**

To assess the impact of knowing central corneal thickness (CCT) on glaucoma management in a United Kingdom district general hospital.

**Methods:**

A masked observational non-interventional study included 304 eyes of 152 consecutive glaucoma cases attending general clinic. CCT was measured using a hand-held pachymeter. IOP, as measured by the Goldmann applanation tonometer (GAT), was adjusted for CCT using a normogram. Two identical study sheets were retrospectively constructed from each subject's case notes: one included the CCT and adjusted IOP information, the other excluded. Study sheets were randomly presented to a single masked observer to decide glaucoma management. The difference in management decision was noted.

**Results:**

The mean ± standard deviation CCT was 561.5 ± 35.7 μm, 538.9 ± 41.4 μm, 538.3 ± 40.3 μm for ocular hypertension (OHT), primary open angle glaucoma (POAG) and normal pressure glaucoma (NPG) subjects respectively. IOP adjustment was greater than ±2 mmHg in 33.9%(103/304) of eyes. CCT and adjusted IOP information led to different treatment option in 37%(55/152). Of the most important changes 20.4%(31/152) cases would have been commenced on additional IOP-lowering medication, 2.0%(3/152) would have been counselled for trabeculectomy surgery and 3.3%(5/152) of the cohort would have been observed rather than treated.

**Conclusion:**

CCT and adjusted IOP measurement can influence glaucoma management in a clinical context. It helps attribute risk and hence aids patient management decisions.

## Background

Measuring intraocular pressure (IOP) is well established, with the Goldmann Applanantion Tonometer (GAT) being the most widely used device. The influence of corneal thickness on IOP by conventional tonometers was acknowledged by Goldmann [1] and clarified later by other investigators [2-6]. It has been recommended by many that GAT readings should be complimented with CCT measurements [2,3]. Normograms, based on varying CCT, exist for adjusting GAT readings in normal eyes [4-6]. There is much controversy regarding these scales and no single one has proven to be satisfactory as the relationship between IOP and CCT is variable [3,7,8]. What is certain is CCT measurement can allow for a more accurate estimate of the true IOP [3,5] and as IOP is the main risk factor for the progression of glaucoma [9-11] using CCT in routine examinations seems mandatory.

Corneal thickness can aid classification in glaucoma suspects between primary open angle glaucoma (POAG), ocular hypertension (OHT) and normal pressure glaucoma (NPG) [12]. In addition measuring CCT was recommended by the ocular hypertension studies (OHTS) [13,14] as it is a predictive factor for the conversion of OHT to POAG. Therefore knowledge of CCT can help to attribute the likelihood of disease progression and assigning the risk can change clinical management decisions to reach a personalized target pressure.

We believe CCT measurement is still not a routine part of eye examinations in some United Kingdom general clinics. The aim of this experiment was to analyse any change in management decisions based on a CCT measurement being revealed to a masked observer. This would help assess the influence of CCT measurements in decision making and gauge the importance of all general ophthalmology clinics having access to a pachymeter.

## Methods

This observational non-interventional experiment studied 304 eyes of 152 consecutive patients who attended a general ophthalmologist's clinics with a diagnosis of glaucoma. For the purpose of this study only cases with NPG, POAG or OHT were included. They were classified in the following way: POAG was defined as IOP ≥22 mmHg, in presence of demonstrable visual field loss and/or significant optic disc cupping. While IOP ≤ 22 mmHg with visual field loss and/or significant disc cupping cases were defined as having NPG. OHT was defined as IOP ≥ 22 mmHg in the absence of visual field loss or significant optic disc cupping. Exclusion criteria included any cases below the age of 18 years, any subjects with ocular co-morbidity such as previous corneal surgery or disease, any subjects with a diagnosis of secondary glaucoma, or where the diagnosis was unknown (i.e. glaucoma suspects).

Local ethics committee approval was obtained for this study. Measurements were only taken after informed consent was taken, and the tenets of the declaration of Helsinki were observed. Inclusion in the study was only after informed consent was taken. Subjects were routinely managed in clinic as normal. They consented for their case history to be reviewed, anonymised, summarised and that information to be used in this study. As is normal procedure in clinic IOP was measured using the GAT with topical proxymetacaine 0.5% and fluorescein (Bausch & Lomb, Rochester, New York, USA) instilled into both eyes. The CCT measurements were recorded from a seated patient using a hand held ultrasonic pachymeter probe (Pachmate™ DGH 55, DGH Technology Inc, PA, USA) gently placed in the mid-pupillary axis of the cornea in the undilated eye. The Pachmate™ gives a mean value of CCT taken from 25 separate measurements. All measurements were taken by three senior ophthalmologists and both the tonometry and pachymetry were only taken once in each subject.

**Figure 1:**
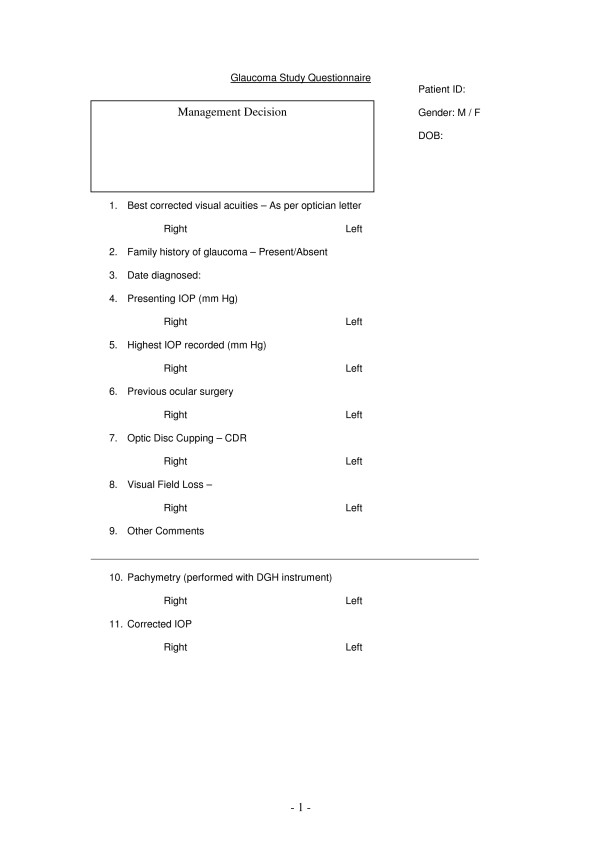
Data collection sheet used in the study.

The IOP adjustment was made according to the manufacturer's logarithm which is based on Ehlers et al. [5], see Table 1. All notes were retrospectively reviewed and summarised by 2 investigators. Two identical study sheets were then constructed: one included the CCT and adjusted IOP information, the other excluding these values (Figure 1 shows an example of a study sheet). The adjusted IOP value was made using the pachymeter manufacturer's algorithm which is based on a cannulation study done by Ehlers et al. [5].

**Table 1:** Correction table used for adjusting IOP based on central corneal thickness (provided with Pachmate™ pachymeter and based on Ehlers et al [5]).

**Central Corneal Thickness (Microns)**	**Adjustment in IOP (mm Hg)**
445	+7
455	+6
465	+6
475	+5
485	+4
495	+4
505	+3
515	+2
525	+1
535	+1
545	0
555	-1
565	-1
575	-2
585	-3
595	-4
605	-4
615	-5
625	-6
635	-6
645	-7

Study sheets were shuffled and randomly presented to a single masked observer (PH) to decide glaucoma management. Using study sheets was an attempt to reduce any bias from memory effects of the assessor between cases. The investigators then recorded any differences in management decisions that had been noted by PH.

### Statistical analysis of data

Excel (Microsoft Corporation, Redmond, WA, USA) was used to analyze and present data. The level of significance was chosen at p < 0.05.

## Results

Of 152 patients studied, 84 were male and 68 were female. The mean age was 71.0 years (±11.5 standard deviation years) POAG, OHT and NPG were classified in 49.3% (150/304), 34.9% (106/304) and 15.8% (48/304) of the cohort, respectively. The mean ± standard deviation (SD) CCT of cohort was 546.7(±40.7) μm. Mean CCT in the POAG group was 538.3(±40.3) μm, OHT eyes it was 561.5 (±35.7) μm and in NPG eyes it was 538.3(±40.3) μm. The difference between all three groups was statistically significant (p < 0.0001, ANOVA test).

**Figure 2:**
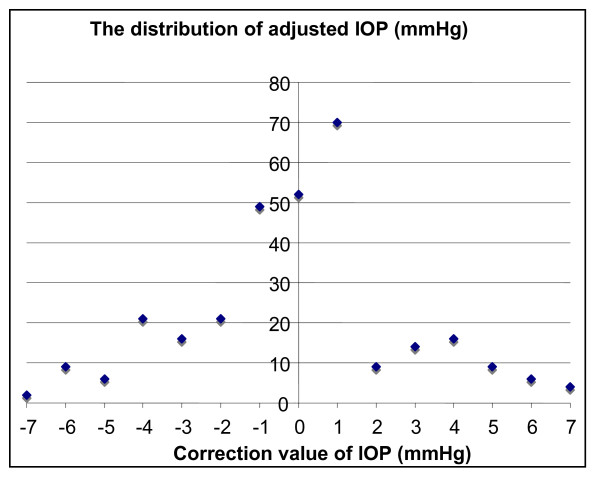
Chart showing the frequency of each level of IOP adjustment in the cohort, based on table 1 (correction of IOP by CCT [5]).

Based on CCT readings, 33.9% (103/304) had the IOP adjusted by greater than ±2 mmHg. 18% (54/304) of eyes had the IOP reduced while 16.1% (49/304) had their IOP increased. Table 2 shows details of these adjustments made in three groups while Figure 2 shows the distribution of the adjustment to be made to IOP

**Table 2:** A summary of IOP adjustment of more than 2 mm Hg according to the diagnosis (n = 103)

	Total number	IOP reduced	IOP increased
POAG	50	19	31
OHT	38	29	9
NPG	15	6	9

Analysis of change in management decision showed that the masked observer subsequently suggested different treatment in 36.2% (55/152) of subjects based on CCT being made available. These differences were divided into 2 categories: when treatment was deemed to be insufficient and upgrading of treatment was suggested (under-treated group) and second, where it was deemed that subjects were over-treated, downgrading of treatment was suggested (over-treated group). Clinical outcomes of the decision change are shown in Table 3.

**Table 3:** A summary of the change in treatment for both the under-and over-treated groups (total cohort n = 152; change in management decision n = 55)

**Under-treatment group (n = 34)**	**Over-treatment group (n = 21)**
31 cases would add additional IOP lowering therapy	11 cases would discharge back to optician
3 cases would council for trabeculectomy	5 cases would observe rather than treat
	5 cases would consider treatment reduction

## Discussion

Clinical decision-making relies on established and quantifiable parameters; these must be used rather than a reliance on medical impression and intuition. The importance of CCT is now well known and a proven parameter [13,14]. Shih et al. [15] had a similar objective: to ascertain whether CCT affected patient management. Their study, although set within a specialist glaucoma service, had similar results which showed that half their study population required an adjustment of IOP ± 1.5 mmHg. What is interesting is that 8–10% of their cohort had a change in their medication, whereas we found nearly one third of ours would have had their treatment changed in light of the CCT and adjusted IOP measurement. This higher proportion may possibly reflect the difference in IOP correcting algorithm.

The use of normograms remains controversial. Gunvant et al. [16] compared three formulae, including Ehler's [5], and concluded that it and the others investigated (Orssengo-Pye) may significantly over estimate the effect of CCT on IOP measurement and lead to an overcorrection of IOP. It is possible that if the duplicate sheets had only contained CCT, instead of both CCT and IOP adjusted for CCT using Ehler's algorithm, the decision made could have been different. It would have relied on the single clinician's own intuition and knowledge, and not on a defined and quantifiable scale. Therefore we feel that although no single algorithm for correcting factors is well accepted; the use of such a scale was justified for this type of experiment.

While this study only tested one senior ophthalmologist's practice, it is clear from the results, that lack of one investigation or parameter such as CCT, can lead to a significantly different outcome for the individual patient: a 6 month review appointment changed to counseling for trabeculectomy surgery in 2% of the cohort. Being a theoretical experiment is a limitation of this study: it could be argued that the decision maker (PH) was in an artificial situation where patients were not actually involved, and this could possibly change his actual decision. However the aim of the study was to provide evidence that knowledge of CCT changed management decisions and in 36% of the cohort it did. Although only one reading of CCT was taken in this study we are aware that it has been suggested that more than one CCT measurements are required, as there can be a significant difference between measurements taken at different times from the same eye [17].

## Conclusion

Implementation of routine central corneal thickness measurement could change patient management in the general ophthalmologist's practise. We feel that a pachymeter is an essential item of the ophthalmic equipment armamentarium. The cost of such an item to a department is small compared to being able to confidently relax or step up a patient's follow-up or treatment.

CCT is one factor that is necessary to adjust IOP to achieve a more accurate IOP and it allows monitoring for the risk of progression to be more precise. Any decision in glaucoma, in the absence of CCT is an uninformed one.

## Competing interests

The author(s) declare that they have no competing interests.

## Authors' contributions

AP, MK and PH conceived and conducted the study. AP and SPM analyzed the study and contributed to the writing of the manuscript. All authors read and approved the final manuscript.

## Pre-publication history

The pre-publication history for this paper can be accessed here:


